# The Role of Secondary Surgery in Recurrent Ovarian Cancer

**DOI:** 10.1155/2012/613980

**Published:** 2012-08-05

**Authors:** D. Lorusso, M. Mancini, R. Di Rocco, R. Fontanelli, F. Raspagliesi

**Affiliations:** Fondazione IRCCS Istituto Nazionale Tumori, Via Venezian 1, 20133 Milan, Italy

## Abstract

Despite optimal treatment (complete cytoreduction and adjuvant chemotherapy), 5-year survival for advanced ovarian cancer is approximately 30% and most patients succumb to their disease. Cytoreductive surgery is accepted as a major treatment of primary ovarian cancer but its role in recurrent disease is controversial and remains a field of discussion mainly owing to missing data from prospective randomized trials. A critical review of literature evidence on secondary surgery in recurrent ovarian cancer will be described.

## 1. Introduction

Despite optimal treatment  (complete cytoreduction and adjuvant platinum-paclitaxel chemotherapy), 5-year survival for advanced ovarian cancer is approximately 30% [1] and most patients succumb to their disease. Overall, 85% of ovarian cancer patients will experience recurrent disease, with virtually no long-term survival after recurrence. Cytoreductive surgery is accepted as a major treatment of primary ovarian cancer but its role in recurrent disease is controversial and remains a field of discussion mainly owing to missing data from prospective randomized trials and to the broad variety of definitions of surgical procedures. Moreover, different studies include different groups of patients ranging from patients with persistent disease at the end of first line treatment (which possibly includes patients with persisting and/or progressing disease at the completion of carboplatin-paclitaxel chemotherapy) to patients with recurrent disease after a disease-free period variable from some weeks to several years [2, 3].

In addition, all but one series are represented by retrospective studies and obviously suffer from selection bias. Generally, the rate of patients not offered secondary surgery at recurrence varied from 7 to 64% among different trials but unfortunately informations about selection criteria and outcomes of nonsurgery selected populations are lacking. 

Moreover, given the long time span of most studies (>5–10 years), the pre- and postoperative chemotherapy treatments varied widely between patients thus increasing the difficulties in the interpretation of data. 

None of the studies details how recurrence was detected, the type of followup adopted after primary treatment, and the selection criteria used for secondary cytoreduction which broadly differ between studies. 

Although the recently published MRC OVO5/EORTC 55955 trial [4] concluded that early intervention with chemotherapy for recurrent ovarian cancer detected only on the basis of serum CA 125 rising does not alter overall survival with respect to waiting for the appearance of symptomatic disease, Tanner et al. [5] found that survival after ovarian cancer recurrence was greater in asymptomatic patients than in those with symptoms (45 versus 29.4 months, *P* = 0.006), and this was due to the rate of successful secondary cytoreductive surgery which was higher in the asymptomatic group (90% versus 57%, *P* = 0.053). Even if retrospective in nature, this study seems to suggest that early surgery in asymptomatic patients with recurrent ovarian cancer may be of benefit thus underling the opportunity of continuous clinical and radiological followup at the end of first line treatment.

Unfortunately, the only prospective randomized trial addressing the role of secondary surgery in recurrent ovarian cancer, the LOROCSON trial, sponsored by European Organization for Research and Treatment of Cancer (EORTC), aborted prematurely due to low recruitment. 

Since the publication by Berek et al. in 1983, which first introduced the term “secondary cytoreduction,” the clinical scenarios and indications of repeated tumor cytoreductive operations for recurrent ovarian cancer have been more precisely defined [21]. According to most clinicians, secondary cytoreductive surgery for recurrent ovarian cancer is defined as an operative procedure performed at some time remote (generally disease free interval of more than 6 months) from the completion of primary therapy with the intended purpose of tumor reduction. Although usually not curative, this kind of surgery aims at prolongation of survival by reducing tumor burden and at improvement of quality of life and cancer-related symptoms. 

The only 2 studies looking at secondary cytoreduction in patients with suboptimal response to primary treatment showed a marginal benefit of surgery at the cost of high morbidity  (24%) and limited long-term benefit with a median survival of 9 months [6, 22] so, at present, there is no evidence that secondary surgery is of significant benefit in this population. 

## 2. Rationale for Surgery

The rationale of surgical removal or recurrent tumor is high and it is supported by the mathematical model of Goldie and Coldman predicting drug resistance in cancer [23] and suggesting that the likelihood of chemotherapy being curable is related to the number of tumor cells present (10^5^  tumor cells are likely to be curable with chemotherapy, but 1 cm tumor nodules contain 10^6^–10^7^ cells). Other theoretical benefits are the removal of a poorly vascularized tumor which may represent pharmacologic sanctuaries of drug resistance; a higher growth fraction in the better perfuse small residual tumor masses which favors the action of cytotoxic therapy; the potentially fewer number of chemotherapy cycles required by small tumor masses limiting the probability of inducing drug resistance; finally the enhancement of host immunocompetence generated by the removal of large tumor bulk.

## 3. Definition of Residual Disease

Almost all series reported a relationship between survival and surgical outcome in univariate analysis and complete debulking is one of the strongest predictors for survival in all multivariate analyses (Table 1). At present what is considered “optimal cytoreduction”? The definition of optimal residual disease widely varies across studies; while some authors argue that optimal cytoreduction can only be described as “absence of visible disease” at the end of the operation, others use less than 0.5 cm [10, 13], less than 1 cm [16, 17, 19, 20], less than 1.5 cm [21], or less than 2 cm cutoff [6–9, 12, 14]. All the studies report superior overall survival in optimally cytoreducted patients, regardless of the discrepancy in how “optimal cytoreduction” was defined. The studies stratifying the subgroup with “absence of visible disease” consistently demonstrate superior results in this group [6, 7, 10–13, 15, 17, 20] with respect to all the other residual disease cutoff groups. 

The large multicenter prospective trial DESKTOP I (Descriptive Evaluation of Preoperative Selection Criteria for Operability) [24] has clearly demonstrated that only complete debulking has prognostic influence and that the “so-called” optimal debulking with residuals up to 1 cm plays no role in surgery for recurrent ovarian cancer. The DESKTOP I trial identified residual disease after surgery as the strongest independent prognostic factor for survival in combination with the absence of ascites and platinum-based reinduction chemotherapy. 

Most studies document approximately 50% of patients being cytoreducted to absence of residual disease, but the complete debulking rate varied from 9% to 85% [13, 16] likely being these differences expression of variances in patients selection criteria, definitions of optimal cytoreduction, surgical techniques, and aggressiveness of surgeons.

## 4. How to Identify Patients Who Most Likely Benefit?

The DESKTOP I trial [24] identified an independently predictive score for complete resection (AGO score) comprehensive of good performance status (Eastern Cooperative Oncology Group 0), complete resection at primary surgery (or alternatively, International Federation of Gynecology and Obstetrics stage I/II), and the absence of ascites. If all the 3 factors were contemporarily present (positive AGO score), complete resection was feasible in 79% of patients. The subsequent international multicenter trial DESKTOP II prospectively validated this score [25]: 129 patients with positive AGO score submitted to secondary surgery for ovarian cancer recurrence were enrolled with a confirmed complete resection rate of 76%. 

Several studies have been published addressing the role of radiological evaluation in predicting successful secondary surgery but all of the series were retrospective evaluations, never prospectively validated [26]. Laparoscopic evaluation of successful surgery was published by an Italian group with percentages of complete resections comparable to what obtained with AGO score but at higher price in terms of complications and feasibility of the procedure [27]. 

None of the published series reported age as a predictor of resectability but most of them excluded patients older than 70 years old from secondary surgery.

The presence of cancer-related symptoms, tumor burden, presurgical serum CA 125 values, localizations of disease, and treatment-free interval (TFI) were inconstantly reported as predictive factors of tumor resectability in univariate and multivariate analysis. 

## 5. Prognostic Factors for Survival

Complete debulking was the strongest predictor for survival in all the multivariate analyses performed across the studies (Table 2). All the other analyzed factors provided conflicting results. Treatment free interval before secondary surgery did not show any significant impact on outcome in univariate analysis in approximately half of the published series and even where reported did not retain independent prognostic significance in multivariate analysis. Of note, a very poor percentage of enrolled patients presented TFI less than 6 months (0–13.5%) suggesting that data addressing a possible impact of TFI on survival are mainly valid for different periods beyond 6 months. 

The absence of ascites and the reintroduction of platinum as adjuvant treatment after surgery are generally associate with prolonged survival.  On the contrary, unfavorable outcome was reported for patients receiving preoperative chemotherapy possibly for the emergence and selections of chemotherapy resistant foci. The impact of preoperative tumor load remains controversial: an exploratory analysis of DESKTOP 1 trial showed that peritoneal carcinomatosis is a significant negative predictor for complete resection, but if complete resection is still possible there is no difference in survival compared to completely debulked patients without peritoneal carcinomatosis [28]. 

## 6. Comparison with Chemotherapy

Platinum-based combinations appear as the most suitable and active treatments for recurrent platinum-sensitive (platinum-free interval >6 months) ovarian cancer patients. Combinations with taxanes [29], gemcitabine [30], and pegylated liposomal doxorubicin [31] reported a median survival of  29, 18, and 31.5 months in the respective superior arms which is generally poorer than the median overall survival reported by the majority of trials of optimally debulked recurrent ovarian cancer patients [32]. Moreover, a meta-analysis on the role of secondary surgery for recurrent ovarian cancer on 40 studies in 2019 patients reported that each 10% increase in optimally cytoreducted patients translates into a 3-month increase of overall survival [33]. 

 Güngör et al. [18] reported in a retrospective review that patients who underwent successful secondary cytoreductive surgery had an improved survival over patients who had chemotherapy as exclusive treatment at recurrence. Unfortunately, in absence of a prospective randomized trial, such conclusions may represent the result of selection bias rather than the effect of surgery.

A recently published Cochrane Review [34] found no evidence from randomized clinical trials to inform decisions about secondary surgical cytoreduction and chemotherapy compared to chemotherapy alone for women with recurrent epithelial ovarian cancer. The author concluded that ideally, a large randomized controlled trial or, at the very least, well-designed nonrandomized studies that use multivariate analysis to adjust for baseline imbalances are needed to compare these two treatment modalities.

The AGO group started with the DESKTOP III trial in Q3 2011. This study is a randomized phase III trial comparing cytoreductive surgery followed by platinum-based chemotherapy versus chemotherapy alone in a population of 408 recurrent platinum sensitive ovarian cancer with positive AGO score at first event of disease recurrence. 

A similar study, the GOG 213 study, is ongoing in the United States addressing two different questions: the role of secondary surgery in recurrent platinum-sensitive ovarian cancer and the inclusion of bevacizumab in combination or not to standard carboplatin-paclitaxel treatment as adjuvant chemotherapy. 

## 7. Morbidity and Quality of Life

Due to the retrospective nature of most studies, reliable information on QOL and postoperative morbidity are often not available. Most studies reported around 30–40% postoperative morbidity with severe morbidity (including sepsis, hemorrhage, adult respiratory distress syndrome, bowel obstruction, and disseminated intravascular coagulation) quoted around 10% and up to 2% postoperative mortality registered [12, 13, 15].

Very little is known about quality of life (QOL) after secondary cytoreduction. Wenzel et al. [35] reported no difference in QOL in a randomized multicenter trial comparing FIGO stage III-IV ovarian cancer patients who did and did not undergo interval debulking surgery after incomplete primary cytoreduction and 3 cycles of platinum-paclitaxel chemotherapy thus suggesting that additional surgical interventions in ovarian cancer may not have any significant impact (positive or negative) on QOL.

## 8. Cytoreductive Surgery and Hyperthermic Intraperitoneal Chemotherapy (HIPEC)

Cytoreductive surgery and HIPEC have yielded promising results in malignant disease for which no other systemic therapies have been shown to be beneficial [36–38]. As far as ovarian cancer is concerned, Chua et al. [39] systematically reviewed the oncologic outcome, morbidity and mortality of cytoreductive surgery, and HIPEC of 19 retrospective observational studies from 10 high volume specialized treatment centers and reported a severe morbidity rate ranging from 12% to 63%, a treatment-related mortality ranging from 0.9% to 10%, and a median overall survival ranging from 22 to 64 months. Such a broad variability in reported oncologic outcomes and toxicities represents the clear expression of the extreme heterogeneity in enrolled population, the different time point of HIPEC administration during the natural history of ovarian cancer (published data are a miscellanea of interval debulking surgery, second-look surgery, and secondary cytoreduction of mixed platinum-sensitive and resistant patients), the variability of chemotherapy employed, the center expertise, and also possibly may represent the expression of a wide different clinicians' interpretation and reporting of data which make it exceptionally difficult to draw definitive conclusions.

The absence of valuable alternative treatment option, as suggested by few authors, is not an acceptable criteria “per  se” for further promoting this concept: the high level of perioperative morbidity and mortality might be considered acceptable when no other alternate therapy has been shown to be effective in curing or controlling the disease but this is not the case of ovarian cancer recurrence in which surgery and platinum-based chemotherapy represent accepted, evidence-based, valuable options.

Ovarian cancer moreover is quite a different disease with respect to colon cancer in which only one randomized trial comparing HIPEC versus palliative surgery plus chemotherapy has ratified HIPEC as the new standard treatment of peritoneal colon cancer carcinomatosis [40], and conclusions derived from one type of malignancy should not be arbitrarily applied to another tumor.

Finally, all the published studies so far are phase I and II feasibility, nonrandomized trials, which makes it difficult to meaningfully compare neither the risk-benefit ratio associated with HIPEC+ cytoreductive surgery (CRS) versus CRS alone (no doubts remain on the usefulness of maximal cytoreduction “per se” among all the investigators who manage ovarian carcinoma), nor the exact role of hyperthermia in combination with intraperitoneal delivery of chemotherapy versus the more established benefit of intraperitoneal chemotherapy alone. The benefit of CRS plus HIPEC depends on all these procedures being carried out in selected patients but no data are available on the relative weight of each of them. 

For all of these reasons, we emphasize that the real survival benefit of HIPEC in ovarian cancer could only be assessed by a well-designed prospective randomized phase III trials. At present, no evidence yet shows that HIPEC benefit in ovarian cancer outweigh the contraindications or risks related to morbidity, mortalitys or costs. At this regard our controlled randomized experience on HIPEC in recurrent ovarian cancer (submitted paper) and Pomel et al. prospective experience [41] registered unacceptable high level of morbidity and mortality causing the premature conclusion of the two trials. 

## 9. Conclusions

Although the role of secondary surgery in recurrent ovarian cancer remains controversial, most retrospective studies showed better survival in patients for whom maximal cytoreduction was achieved. Due to the retrospective nature of these studies, multiple confounding factors play a role in selection and operability of these patients; moreover, at present, the indications for surgery when a recurrence is diagnosed appear more often dependent on the physician's preference and surgical skill than on patients' attitudes or tumor characteristics. 

We strongly believe that there is a role for secondary cytoreductive surgery in a well-selected population.  At present, the main recognized factors improving the likelihood of optimal secondary cytoreduction and possibly contributing to prolong patients survival are the absence of ascites, a good performance status, and the complete debulking during primary surgery (AGO score). A better understanding of the benefits and patients selection criteria for this procedure will be achieved after the completion of the ongoing randomized phase III trials evaluating the role of surgery for recurrent disease. A positive outcome of these trials will lead to the addiction of this strategy to the standard armamentarium therapies of ovarian cancer recurrence.

## Figures and Tables

**Table 1:** Published results on secondary cytoreduction for recurrent ovarian cancer.

Reference	*n*	Definition used	Cytoreduced (%)	Median survival after secondary surgery
				(months)
Berek [3]	21	RD<o>1.5 cm;	6/21 (29); 15/21 (71)	<1.5, (20); >1.5 (5) *P* < 0.01
Morris [6]	30	NVD; RD<o>2 cm	9/30 (30); 8/30 (27); 13/30 (43)	<2 (18.8); > 2(13.3) ns
Janicke [7]	28	NVD; RD<o> 2 cm	14/28 (50); 12/28 (43); 2/28 (7)	NVD, (29); RD, (9) *P* = 0.0000
Segna [8]	100	RD<o>2 cm	61/100 (61); 39/100 (39)	<2,(27.1); >2, (9) *P* = 0.0001

			3/5 stage I (60);	
Pecorelli [9]	270	NVD; RD RD<o>2 cm	2/5 stage I (40);	<2 (20); >2 (12) *P* = 0.045
			13/22 stage III (58);	
			9/22 stage III ( 41)	

Vaccarello [10]	57	RD<o>0.5 cm	38/57 (67); 23/38	<0.5, NYR; >0.5 (23)
Cormio [11]	21	NVD; no cytoreduction	15/21 (71); 6/21 (25)	NVD, (32); RD (9) *P* = 0.029)
Gadducci [12]	30	NVD RD<o>2 cm	17/30 (57); 8/30 (27); 5/30 (17)	NVD (37); RD (19) *P* = 0.04
Eisenkop [13]	106	NVD; RD<o>2 cm	87/106 (82); 3/106 (3); 16/106 (15)	NVD (44.4); RD (19.3) *P* = 0.0007
Munkarah [14]	25	NVD; RD<o>2 cm	12/25 (48); 6/25 (24); 7/25 (28)	NVD (56.9); RD (25.1) *P* = 0.08
Tay [15]	46	NVD; RD	19/46 (41); 27/46 (59)	NVD (38); RD (11) *P* = 0.002
Zang [16]	107	NVD; RD<o>1 cm	11/107 (10); 61/107 (57); 35/107 (33)	NVD nyr; RD < 1 (26); >1(14.5)
Onda [17]	44	NVD; RD<o>1 cm	26/44 (59); 11/44 (25); 7/44 (16)	NVD (52); RD<1 (23) *P* = 0.0007; >1 (20)
Güngör [18]	44	Surgery; chemo only; NVD	44/75 (59); 31/75 (41); 34/44 (77)	NVD (19); RD (9) *P* = 0.007
Pfisterer [19]	267	NVD; RD<o>1 cm	133/267 (50); 69/267 (26); 65/267 (24)	NVD (45.3); RD (19) *P* < 0.0001
Ayhan [20]	64	NVD; RD<o>1 cm	28/64 (44); 25/64 (39); 11/64 (17)	<1 (28); >1 (18) *P* = 0.004

RD: residual disease, NVD: no visible disease, NS: not significant, NYR: not yet reached.

**Table 2:** Multivariate analysis for survival.

	Zang	Eisenkop	Tay	Zang	Onda	Pfisterer	Ayhan
	[16]	[13]	[15]	[16]	[17]	[19]	[20]
*n*	60	106	46	107	44	267	64
Age		Ns		Ns	Ns		Ns
PS		Ns			Ns		Ns
Initial FIGO	Ns		Ns	Ns	Ns		Ns
Grade	Ns	Ns	Ns				Ns
Histology	Ns			0.017	Ns		0.005
RD after primary surgery				Ns	Ns		0.003
DFI	0.0116	0.005	0.001			<0.001	0.003
RD after secondary surgery	0.0041	0.007	0.002			<0.001	0.04
Disease localization		Ns			0.013		
No disease sites					<0.001		
Largest tumor diameter	Ns	0.04			<0.001		
Ascites^1^	0.0191	Ns			Ns	0.012	
Ca 125^1^		Ns					
No cycles chemo^2^	Ns						
Chemo^2^	Ns	0.001					

Ns: not significant, RD: residual disease, DFI: disease-free interval.

^1^At secondary surgery.

^2^Prior to secondary surgery.
